# miR136 regulates proliferation and differentiation of small tail han sheep preadipocytes

**DOI:** 10.1080/21623945.2023.2173966

**Published:** 2023-02-10

**Authors:** Man Luo, Lin Wang, Cheng Xiao, Mengsi Zhou, Minghui Li, Hongjuan Li

**Affiliations:** a Metabolic Disease Research Center, Zhengzhou Central Hospital Affiliated to Zhengzhou University, Zhengzhou, China; b Department of Obstetrics and Gynecology, Zhengzhou Central Hospital Affiliated to Zhengzhou University, Zhengzhou, China; c School of Basic Medical Sciences, Zhengzhou University, Zhengzhou, China; d Institute of Animal Biotechnology, Jilin Academy of Agricultural Sciences, Gongzhuling, China

**Keywords:** miR136, differentiation, proliferation, sheep preadipocytes, intramuscular fat

## Abstract

Low meat performance is the defect of Small Tail Han sheep. Intramuscular fat affects meat quality and largely determined by adipogenesis. In previous study, miR136 was showed one of differentially expressed microRNAs between preadipocytes and mature adipocytes of Small Tail Han sheep but its role in adipogenesis is still not elucidated. Here, we investigated the effect of miR136 on adipogenesis and the underlying mechanism. qPCR data showed that miR136 level increased with preadipocytes proliferation while declined with preadipocytes differentiation. Moreover, miR136 mimics blocked lipid droplet formation, reduced lipid content and triglyceride accumulation while miR136 inhibitor showed the opposite effects, revealing that miR136 promoted preadipocytes proliferation but inhibited preadipocytes differentiation. Bioinformatics and biochemical validation manifested that PPARGC1B was a target of miR136. Furthermore, miR136 mimics decreased PPARγ and C/EBPα expression accompanied by PPARGC1B expression descending. Reverse effects were observed with miR136 inhibitor. Besides, overexpression of miR136 elevated IGF1 expression. Collectively, our data first exhibited a regulatory role of miR136 in adipogenesis, which is promoting preadipocytes proliferation through elevating IGF1 expression while inhibiting preadipocytes differentiation through targeting PPARGC1B and further declined PPARγ and C/EBPα expression. The modulation of PPARGC1B by miR136 may provide a new potential target for increasing intramuscular fat.

## Introduction

As a Chinese endemic breed, Small Tail Han sheep possesses high fecundity and strong resistance but low meat performance. Intramuscular fat (IMF) is one of the most critical parameters affecting meat quality. A high percentage of mature adipocytes, some preadipocytes, and a few other cells make up IMF [1]. Accordingly, the accumulation of IMF is largely determined by the process of adipogenesis. During the process of adipogenesis, preadipocytes differentiate into lipid-laden and insulin-responsive mature adipocytes [2].

Adipogenesis involves a complex and highly orchestrated gene expression programme which is controlled by a cascade of transcription factors (TFs) [3]. Insulin like growth factor 1 (IGF1) is an important growth factor and well-known for promoting cellular proliferation [4,5]. As a strong activator of mitochondrial biogenesis and respiration, PPARG coactivator 1 beta (PPARGC1B) generally expressed in tissues with high oxidative capacity such as adipose tissue [6]. The main regulatory mechanism of PPARGC1B is to activate specific target TFs including proliferator-activated receptor gamma (PPARγ) or oestrogen related receptor alpha (ERRα) to give rise to cascade responses downstream. PPARγ is undoubtedly considered as the most important transcriptional modulator of adipocyte development in all types of adipose tissue [7]. Adipogenic stimuli induce terminal differentiation in committed preadipocytes through the epigenomic activation of PPARγ. Furthermore, the coordination of PPARγ with CCAAT enhancer binding protein alpha (C/EBPα) are considered as the vital determinants for adipocyte fate [8]. C/EBPα is critical in the establishment of insulin sensitivity and also maintains PPARγ expression in developing adipocytes. PPARγ and C/EBPα are gene markers of preadipocytes differentiation in the process of adipogenesis [9].

MicroRNAs were reported to play important roles in adipogenesis [10–12], however, there are still a large number of microRNAs have not been studied. In previous study, we compared differences between preadipocytes and mature adipocytes of Small Tail Han sheep by whole-transcriptome sequencing. The expression of miR136 was significantly up-regulated in mature adipocytes compared to preadipocytes. 10 differentially expressed microRNAs (DE miRNAs) were selected to validate the accuracy of RNA-Seq data according to expression level and the core regulatory networks. Among them, the qPCR results of miRNA136 had the strongest consistency with RNA-seq data. Besides, miR136 exhibit the most memorably expression trend in the process of preadipocytes turning to mature adipocytes [13]. miR136 was a newly discovered microRNA and its binding sequences were highly conserved among sheep, cattle and human, however, its function in adipogenesis still remains to be elucidated.

The purpose of this study was to investigate the effect of miR136 on adipogenesis. We analysed the role of miR136 in the proliferation and differentiation of preadipocytes and explored its regulatory mechanism involved in the function of PPARGC1B, PPARγ, C/EBPα and IGF1. Our data indicated that miR136 promoted preadipocytes proliferation through elevating IGF1 expression while inhibited preadipocytes differentiation through directly targeting PPARGC1B and thereby affecting PPARγ and CEBPα expression.

## Results

### miR136 expression pattern in the process of adipogenesis

**Figure 1.**
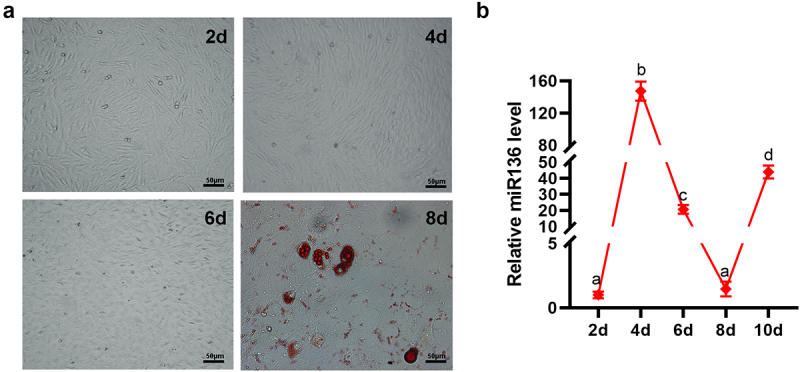
miR136 expression pattern in the process of adipogenesis. (a) Sheep preadipocytes and oil red O staining. The red dots are lipid droplets inside the cell stained red by oil red O. Scales bar: 50 µm. (b) miR136 expression patterns in adipogenesis. Data are presented as ‘mean ± SD’. Different lowercase letters at the top of each bar denote significant differences among groups. The difference among groups was compared by one-way ANOVA with Tukey’s *post hoc* test, P < 0.05.

To investigate the role of miR136 in adipogenesis, preadipocytes were isolated from subcutaneous white adipose tissues of Small Tail Han sheep and induced to mature adipocytes in vitro. As shown in Figure 1a, the intracellular lipid droplets were stained red by oil red O, revealing that the preadipocytes successfully differentiated to mature adipocytes at 8d. qPCR was used to detect miR136 expression pattern in the process of adipogenesis. miR136 level increased along with time during preadipocyte proliferation phase (2d to 4d) while decreased along with time during preadipocytes differentiation (4d to 6d). After the preadipocytes turned to mature adipocytes at 8d, miR136 level ascended again (Figure 1b). These data indicated miR136 may promote the proliferation while inhibit the differentiation of preadipocytes.

### miR136 promoted proliferation but inhibited differentiation of preadipocytes

**Figure 2.**
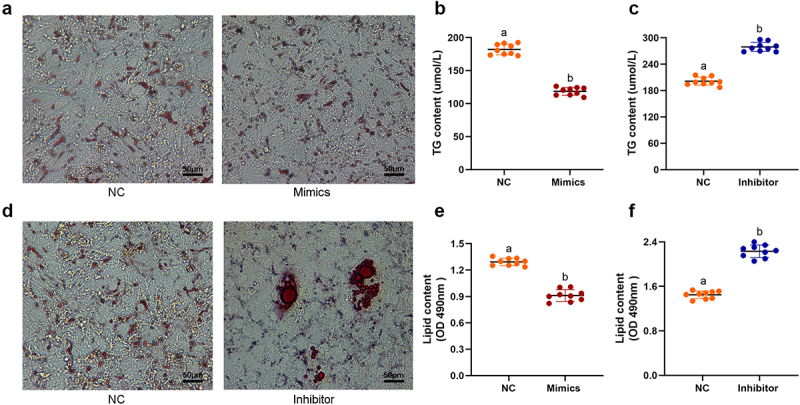
miR136 promoted proliferation but inhibited differentiation of preadipocytes. (a) Sheep preadipocytes and oil red O staining. The red dots are lipid droplets inside the cell stained red by oil red O. Scales bar: 50 µm. (b and e) TG content in preadipocytes after different treatment. (c and f) Lipid content in preadipocytes after different treatment. Data are presented as ‘mean ± SD’. Different lowercase letters at the top of each bar denote significant differences among groups. The difference between groups was compared by Student’s t-test of unpaired data, P < 0.05.

To confirm the role of miR136 in adipocytes, the preadipocytes were transfected with miR136 mimics, inhibitor or negative control (NC). As shown in Figure 2a, lipid droplet formation, which is the signs of mature adipocyte, were hampered by miR136 mimics. The introduction of miR136 mimics abolished preadipocytes differentiation, decreased lipid content and reduced triglyceride content (TG) accumulation (Figure 2a-c). But miR136 inhibitor promoted the differentiation of preadipocytes and significantly elevated the lipid content and TG accumulation (Figure 2d-f). These data confirmed the role of miR136 in adipogenesis which is promoting preadipocytes proliferation but inhibiting preadipocytes differentiation.

### miR136 depressed PPARGC1B expression to inhibit preadipocytes differentiation

**Figure 3.**
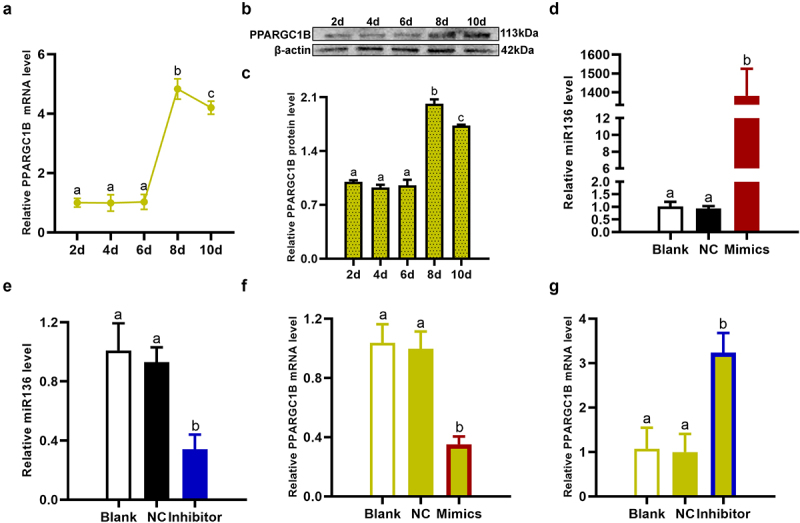
miR136 depressed PPARGC1B expression to inhibit preadipocytes differentiation. (a-c) PPARGC1B expression pattern in adipogenesis. (d and e) miR136 level in preadipocytes after different treatment. (f and g) PPARGC1B mRNA expression in preadipocytes after different treatment. Data are presented as ‘mean ± SD’. Different lowercase letters at the top of each bar denote significant differences among groups. The difference among groups was compared by one-way ANOVA with Tukey’s *post hoc* test, P < 0.05.

Preadipocytes differentiation is the most critical process in adipogenesis, so we first investigated the regulatory mechanism of miR136 in preadipocytes differentiation. As a key coactivator in modulating adipogenesis activities and PPARγ expression [6,7], the level of PPARGC1B was measured by qPCR and Western Blot. As shown in Figure 3a-c, the mRNA expression and protein level of PPARGC1B in cells were very low during the proliferation phase and the first induced differentiation stage of preadipocytes (2d to 6d). However, the mRNA expression and protein level of PPARGC1B were noteworthly increased during the second induced differentiation stage (6d to 8d). After the preadipocytes turned to mature adipocytes at 8d, the mRNA expression and protein level of PPARGC1B descended. Of note, PPARGC1B showed the opposite expression trend to miR136 from 6d to 10d. During preadipocytes differentiation phase (6d to 8d), PPARGC1B presented a notable elevation while miR136 level remarkably went down. However, the expression of PPARGC1B roll back while miR136 level went up after preadipocytes successfully turned to mature adipocytes (8d to 10d). These data suggested that miR136 may depress PPARGC1B to inhibit preadipocytes differentiation.

To further detect the regulator mechanism of miR136 on PPARGC1B, miR136 mimics, miR136 inhibitor and NC were respectively transfected into preadipocytes. The transfection efficiency was verified by qPCR for 48 hours (Figure 3d and e). Overexpression of miR136 significantly brought down the expression of PPARGC1B in preadipocytes (figure 3f). Opposite effect was observed in miR136 inhibitor-treated preadipocytes (Figure 3g), demonstrating the suppression of miR136 on PPARGC1B.

### miR136 inhibited PPARGC1B expression via directly binding to 3′UTR

**Figure 4.**
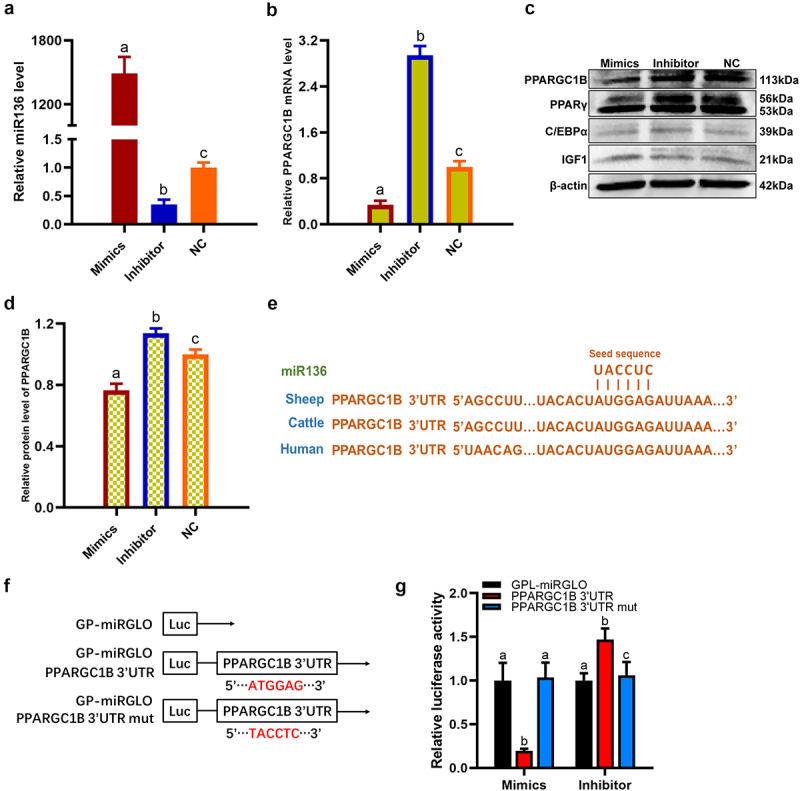
miR136 inhibited PPARGC1B expression via directly binding to 3′UTR. (a) miR136 level in 293 T cells after different treatment. (b) PPARGC1B mRNA expression in 293 T cells after different treatment. (c) The protein expression of PPARGC1B, PPARγ, C/EBPα and IGF1 in 293 T cells after different treatment. (d) PPARGC1B protein level in 293 T cells after different treatment. (e) Sequence alignment of miR136 on the 3′ UTR of PPARGC1B from different organisms. (f) Schematic diagram showing miR136 binding sites and mutation in PPARGC1B 3ʹUTR. (g) Regulation of PPARGC1B via miR136 targeting its 3′ UTR was detected with luciferase reporter assays in 293 T cells. Data are presented as ‘mean ± SD’. Different lowercase letters at the top of each bar denote significant differences among groups. The difference among groups was compared by one-way ANOVA with Tukey’s *post hoc* test, P < 0.05.

Consistent with the data in preadipocytes, qPCR and western blot results showed that PPARGC1B level was enhanced by miR136 inhibitor but declined by miR136 mimics in 293 T cells (Figure 4a-d). The prediction analysis revealed that miR136 binding sites presented in the 3ʹUTR region of the PPARGC1B gene. Moreover, multiple sequences alignment revealed that the potential binding sites of miR136 in the 3ʹUTR of PPARGC1B were highly conserved among human, sheep and cattle (Figure 4e). Accordingly, dual-luciferase reporter vectors of wild-type PPARGC1B 3ʹUTR and mutant PPARGC1B 3ʹUTR were constructed and respectively transfected to 293 T cells (figure 4f). As showed in Figure 4g, miR136 mimics remarkably reduced the luciferase activity of wild-type PPARGC1B 3ʹUTR reporter but was unable to lower the luciferase activity of mutant PPARGC1B 3ʹUTR reporter. miR136 inhibitor significantly elevated the luciferase activity of the wild-type PPARGC1B 3ʹUTR reporter but has no ability to heighted the luciferase activity of mutant PPARGC1B 3ʹUTR reporter. These results manifested that miR136 inhibited PPARGC1B expression via directly binding to 3′UTR.

### miR136 modulated PPARγ and C/EBPα expression via regulating PPARGC1B

**Figure 5.**
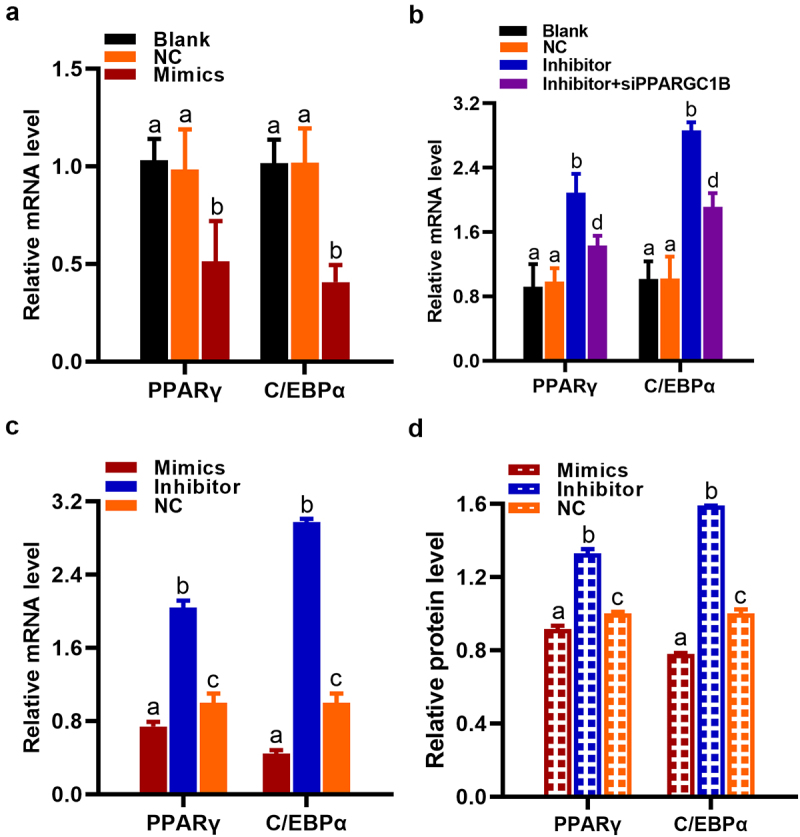
miR136 modulated PPARγ and C/EBPα expression via regulating PPARGC1B. (a and b) PPARγ and C/EBPα mRNA expression in preadipocytes after different treatment. (c and d) The mRNA expression and protein level of PPARγ and C/EBPα in 293 T cells after different treatment. Data are presented as ‘mean ± SD’. Different lowercase letters at the top of each bar denote significant differences among groups. The difference among groups was compared by one-way ANOVA with Tukey’s *post hoc* test, P < 0.05.

PPARγ and C/EBPα are gene markers of preadipocyte differentiation in the process of adipogenesis. PPARGC1B activates PPARγ to give rise to cascade responses downstream. Meanwhile, C/EBPα maintains PPARγ expression in developing adipocytes [7–9]. Therefore, we evaluated the expression of PPARγ and C/EBPα in preadipocytes and 293 T cells after different treatment. As expected, the decline of PPARGC1B expression decreased PPARγ and C/EBPα mRNA expression, accompanied by the abolishment of preadipocytes differentiation (Figure 5a). The enhancement of PPARGC1B expression significantly heightened PPARγ and C/EBPα mRNA expression along with the successfully transition of preadipocytes into mature adipocytes. However, PPARGC1B siRNA hindered these ascents (Figure 5b). In accordance with this, the mRNA expression and protein level of PPARγ and C/EBPα declined together with PPARGC1B expression depression in miR136 mimics-treated 293 T cells compared to NC while miR136 inhibitor showed the opposite effect (Figure 4c, 5c and d).

### miR136 promoted preadipocytes proliferation through elevating IGF1 expression

**Figure 6.**
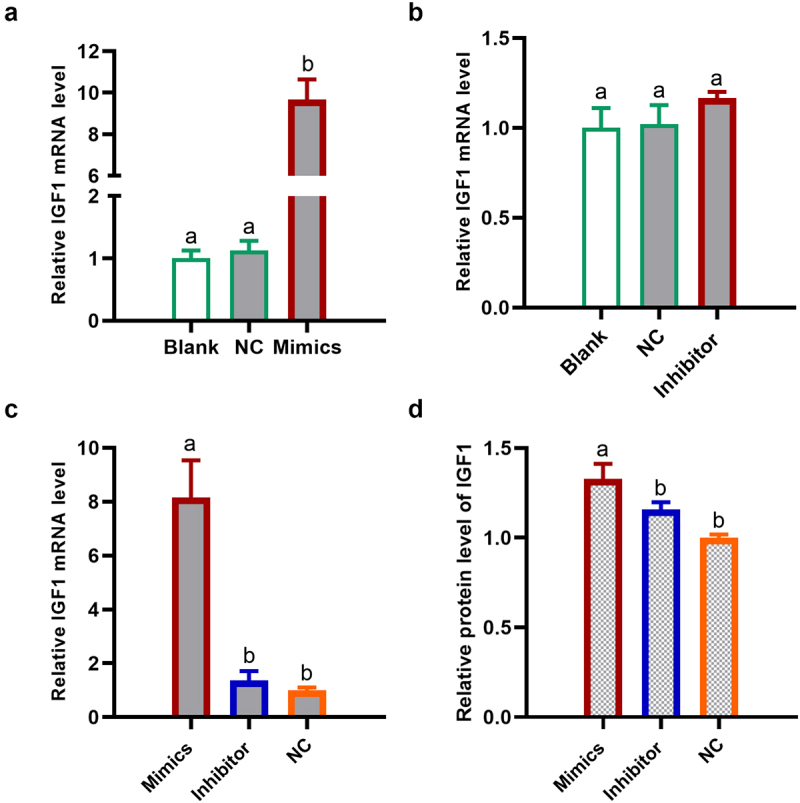
miR136 promoted preadipocytes proliferation through elevating IGF1 expression. (a and b) IGF1 mRNA expression in preadipocytes after different treatment. (c and d) The mRNA expression and protein level of IGF1 in 293 T cells after different treatment. Data are presented as ‘mean ± SD’. Different lowercase letters at the top of each bar denote significant differences among groups. The difference among groups was compared by one-way ANOVA with Tukey’s *post hoc* test, P < 0.05).

IGF1 is well-known for its role in promoting cellular proliferation [5,14]. To explore the regulator mechanism of miR136 on preadipocytes proliferation, IGF1 expression was tested in preadipocytes and 293 T cells. As shown in Figure 6a, the transfection of miR136 mimics remarkably elevated IGF1 mRNA expression in preadipocytes. Although the increase is not significant, miR136 inhibitor also upregulated IGF1 mRNA expression (Figure 6b). Further analysis in 293 T cells showed that the mRNA expression and protein level of IGF1 were significantly ascended by miR136 mimics compared to NC group and miR136 inhibitor group (Figure 4c, 6c and d).

## Discussion

MicroRNAs are small endogenous RNAs that regulate gene-expression post-transcriptionally. In recent years, microRNAs research is expanding since they are crucial regulators of gene expression and promising candidates for biomarker development. In our previous research, the whole-transcriptome analysis and qPCR validation showed that miR136 was one of the significantly up-regulated miRNAs in mature adipocyte compared to preadipocytes [13], therefore we speculated that miR136 may play a role in adipogenesis. As expected, we found that miR136 promoted preadipocytes proliferation while inhibited preadipocytes differentiation. Mechanistically, miR136 promoted preadipocytes proliferation through elevating IGF1 expression. miR136 inhibited the differentiation of preadipocytes by directly suppressing PPARGC1B expression and thereby hampering PPARγ and C/EBPα activation.

PPARγ and C/EBPα are widely accepted as two essential transcription factors (TFs) of preadipocyte differentiation. They work with other TFs to activate genes required for terminal differentiation of preadipocytes both in vitro and in vivo [15]. C/EBPα acts in concert with PPARγ to establish phenotypes of mature adipocytes but cannot function efficiently without PPARγ. In the present study, the transfection of miR136 mimics abated PPARγ and C/EBPα levels and accompanied by the failure of preadipocytes turning to mature adipocytes. Conversely, miRNA136 inhibitor promoted preadipocytes differentiating to mature adipocytes and heightened PPARγ and C/EBPα levels. In line with other reports, these observations revealed that miR136 exerted its function through PPARγ and C/EBPα.

Multiple mechanisms are involved in the activation of PPARγ and C/EBPα, among which PPARGC1B was considered as an important one [6,16]. PPARGC1B is an ‘agonist’ of PPARγ, so we first investigated whether PPARGC1B was a direct target of miR136 or not. The expression trends of miR136 and PPARGC1B were negatively correlated, suggesting that miR136 may directly depress PPARGC1B expression. MicroRNAs were reported to typically suppress gene expression at post-transcriptional levels by directly binding to the 3′UTR of target mRNAs. Similarly, our analysis showed that miR136 depressed PPARGC1B expression via binding to the 3’ UTR region of PPARGC1B. When preadipocytes differentiation began, the decline of miR136 level relieved its suppression on PPARGC1B. Then PPARGC1B activated the PPARγ and C/EBPα expression, giving rise to cascade responses downstream for differentiation.

IGF1 was a crucial functional gene related to proliferation [17,18]. However, the role of IGF1 in adipocytes differentiation is still not defined. In some literature, IGF1 stimulates adipocytes differentiation once growth arrest occurs at confluence [19]. Obesity induces adipocyte stress and increases lipolysis [20] while IGF1 (in vitro) regulates differentiation, promotes survival and suppresses lipolysis of adipocytes [21,22]. In our study, IGF1 level was enhanced by miR136 mimics with the proliferation of preadipocytes. Surprisingly, miR136 inhibitor also enhanced IGF1 level even if the difference is not significant. A possible explanation for this might be that IGF1 is extensively involved in signalling pathways that promote both cell proliferation and cell differentiation, which still need further study [23,24]. Based on our findings, it remains a challenge to uncover the exact role of microRNAs in IMF accumulation. A comprehensive investigation on the transcriptional regulation, post-transcriptional regulation, translational regulation and post-translational regulation would be appropriate for a future study to fully understand the underlying intricacies.

**Figure 7.**
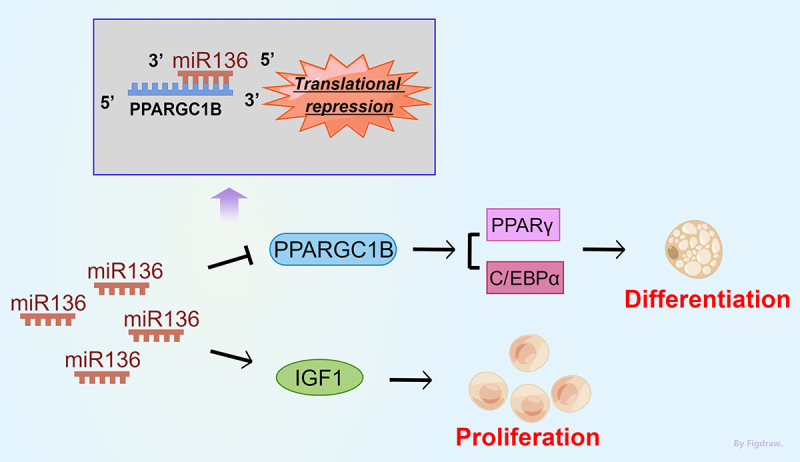
Illustrative model of miR136 role in adipogenesis. miR136 acts as a regulator in the process of adipogenesis. miR136 promoted preadipocytes proliferation via elevating IGF1 expression. miR136 inhibited preadipocyte differentiation by directly targeting PPARGC1B and thereby affecting the expression of PPARγ and C/EBPα.

Taken together, miR136 acts as a regulator in the process of adipogenesis. miR136 promoted preadipocytes proliferation via elevating IGF1 expression. miR136 inhibited preadipocyte differentiation by directly targeting PPARGC1B and thereby affecting the expression of PPARγ and CEBPα (Figure 7). The present study highlights the effect of miR136 in preadipocytes differentiation by modulating PPARGC1B, which may provide a new potential target for increasing IMF content in sheep.

## Materials and methods

### Animals

Male Small Tail Han sheep (1-month-old) were provided by the Institute of Animal Biotechnology (Jilin Academy of Agricultural Sciences, Changchun, China) to separate the preadipocytes. All animal experimental procedures were examined and approved by the Animal Welfare and Ethics Committee of Jilin Academy of Agricultural Sciences (AWEC2019A05).

### Cell isolation, culture, and differentiation

Preadipocytes were isolated from subcutaneous white adipose tissues in sheep groin. The subcutaneous adipose tissues were cut into small pieces and digested by 0.2% collagenase II (Sangon Biotech, A004174) in a 15 mL tube at 37°C for 80 min under aseptic conditions. Subsequently, the digestion products were filtered using 200 and 400 mesh filters, and then were centrifuged three times for 5 min at 1,500 rpm to obtain preadipocytes. Preadipocytes were cultured in petri dishes with complete culture medium including DMEM/F12 medium, 10% (foetal bovine serum) FBS and 1% penicillin-streptomycin at 37°C in 5% CO_2_ incubator. The complete medium was replaced every 48 h. To induce differentiation, exogenous inducer 1 and inducer 2 were added sequentially to the medium after cell overgrowth in petri dishes [13,25]. The inducer 1 consisted of 10 mg/ml insulin (Sigma, I5523), 1.0 mM dexamethasone (Sigma, D4902), 0.5 mM 3-Isobutyl-1-methylxanthine (Sigma, I5879), and complete culture medium. The inducer 2 included 10 mg/ml insulin (Sigma, I5523) and complete culture medium. The cells were treated with inducer 1 for 48 h and then treated with inducer 2 for 48 h. Subsequently, the inducer 2 was altered to complete culture medium and replaced every 48 h. 293 T cells (NSTI-BMCR, 4201HUM-CCTCC00187) were routinely cultured in DMEM/F12 medium supplemented with 10% FBS and 1% penicillin-streptomycin and used to estimate the binding between miR136 and PPARGC1B.

### Oil red O staining

Oil red O staining (Sangon Biotech, E607319) was used to detect the maturation of adipocytes as described previously [25]. Briefly, cells were washed three times with PBS and then fixed with 4% paraformaldehyde in 37°C incubator for 30 min. Subsequently, cells were washed with PBS and stained with oil red O solution at 37°C incubator for 30 min. After that, cells were imaged with a microscope for analysis. Lipid droplets were dissolved by Isopropyl alcohol and the content was calculated at 490 nm using microplate reader.

### Detection of triglyceride content

TG content was detected by EnzyChrom^TM^ Triglyceride Assay Kit (BioAssay System, ETGA-200) according to the kit instruction. The cells were solubilized in 5% Triton X-100 and transferred into separate wells of 96-well plate. Then working reagent was prepared and added into both of standard and sample wells. OD values were calculated at 570 nm after incubation 30 min at room temperature.

### Synthesis of miR136 mimics and inhibitor

miR136 mimics and inhibitor were designed according to miR136 sequences which was obtained from miRBase (http://www.mirbase.org) [26]. miR136 mimics, inhibitor and NC were synthesized by GenePharma Company (C09002, C09003).

### Cell transfection

When the preadipocytes reached approximately 60–70% confluence in six-well plates, mimics (6 µL), inhibitor (6 µL), or NC (6 µL) were respectively transfected to the cells with FuGene HD Transfection Reagent (10 µL) (Promega, E2311) according to the manufacturer’s instructions.

### RNA interference

The small-interfering RNA (siRNA) duplexes for targeting PPARGC1B as well as a scrambled sequence (control siRNA duplex, negative control) were designed and synthesized by GenePharma Company (A10001). Transfections for siRNA were performed by Lipofectamine RNAiMAX transfection reagent (Invitrogen, 13,778,030) according to the instructions of the manufacturer.

### Vector construction and luciferase assays

Bioinformatics websites TargetScan (http://www.targetscan.org) [27] and miRBase [26] were used to predict the binding sequences of miR136 in PPARGC1B 3ʹUTR. The predicted binding sequences of miR136 and corresponding mutant sequences were synthesized and inserted into GP-miRGLO vector (GenePharma, C08006). 293 T cells were co-transfected with appropriate GP-miRGLO constructs and miR136 mimics/inhibitor/NC. Luciferase activity was detected using a dual luciferase reporter gene assay kit (Beyotime, RG042M) as described previously [28].

### RNA extraction and qPCR

To explore the expression patterns of miR136 and PPARGC1B in adipogenesis, total RNA from cells were extracted using TRIzol reagent (Thermo Fisher Scientific, 15,596,018) at 2d, 4d, 6d, 8d and 10d, respectively. To determine the effect of miR136 on preadipocytes proliferation and differentiation, total RNA was extracted after cell transfection 48 h. RNA quality was detected by 1% agarose gels, and the concentration was measured using a Quawell Q5000 spectrophotometer (Quawell Technology, Q5000). Next, total RNA was reversed into cDNA via PrimeScript™ RT reagent Kit. qPCR was used to detect mRNA level using LightCycler® 480 SYBR Green I Master (Roche Applied Science, 04707516001) and performed on Roche LightCycler® 480. Glyceraldehyde-3-Phosphate dehydrogenase gene (GAPDH) and U6 were used as internal controls [13,25]. The reaction conditions were 40 cycles of 95°C for 5 min, 95°C for 10s, 60°C for 15s, and 72°C for 20s, followed by 95°C for 5s, 65°C for 1 min, and 40°C for 10s [19]. The relative expression level of RNAs was calculated using the 2^−ΔΔCt^ method. The primer sequences were described in Table 1.

**Table 1.** The primer sequences of qPCR.

RNAs	Oligo	Primer sequence	Product size	GenBank No.
PPARGC1B	Forward Primer	5’ TGCCCTGATGACTTGGAGC 3’	175bp	XM_027970395.2
	Reverse Primer	5’ CAGAGGAGGAGACGGGTGATG 3’		
PPARγ	Forward Primer Reverse Primer	5’ CCGTGGACCTTTCTATGATGG 3’ 5’ TACAGGCTCCACTTTGATTGC 3’	193 bp	NM_001100921.1
C/EBPα	Forward Primer Reverse Primer	5’ AAGCCAAGAAGTCCGTGGAC 3’ 5’ AGCACCTTCTGTTGCGTCTCC 3’	126 bp	NM_001308574.1
miR136	Forward Primer	5’ ACTCCATTTGTTTTGATGATGGA 3’		
IGF1	Forward Primer Reverse Primer	5’ CAGTCACATCCTCCTCGCA 3’ 5’ TACATCTCCAGCCTCCTCAG 3’	249 bp	NM_001009774.3
GAPDH	Forward Primer Reverse Primer	5’ TCCACGGCACAGTCAAGG 3’ 5’ CACGCCCATCACAAACAT 3’	228 bp	NM_001190390.1
U6	Forward Primer Reverse Primer	5’ CGCTTCGGCAGCACATATAC 3’ 5’ AAATATGGAACGCTTCACGA 3’		

### Western blot

The protein from cells were extracted and quantified by BCA Kit (Beyotime Biotechnology, P0012S) according to the manufacturer’s instruction. The samples were subjected to SDS-PAGE and subsequently transferred to PVDF membranes. Antibodies against PPARγ (1:1000, Bioss, bs-0530 R), C/EBPα (1:1000, Bioss, bs-24,540 R), IGF1 (1:1000, Bioss, bs-0014 R), PPARGC1B (1:1000, Bioss, bs-7534 R) or β-actin (1:5000, Bioss, bsm-33,036 M) were incubated with the membranes at 4°C overnight with constantly shaking. Then the membranes were treated with an HRP-conjugated secondary antibody. The signal was visualized with an enhanced chemiluminescence substrate.

### Statistical Analysis

All experiments were independently repeated at least three times. The data were presented as the mean ± SD and analysed by Student’s t-test of unpaired data or one-way ANOVA with Tukey’s post hoc test using the SPSS 26.0 software programme. P < 0.05 were considered as significant difference.

## Supplementary Material

Supplemental Material

## Data Availability

The data that support the findings of this study are available from the corresponding author juanhongli2022@126.com upon reasonable request.
