# Supplementary figures and images for: miR136 regulates proliferation and differentiation of small tail han sheep preadipocytes
Source: Adipocyte. 2023 Feb 10;12(1):2173966. doi: 10.1080/21623945.2023.2173966 (PMC9928478; doi:10.1080/21623945.2023.2173966)

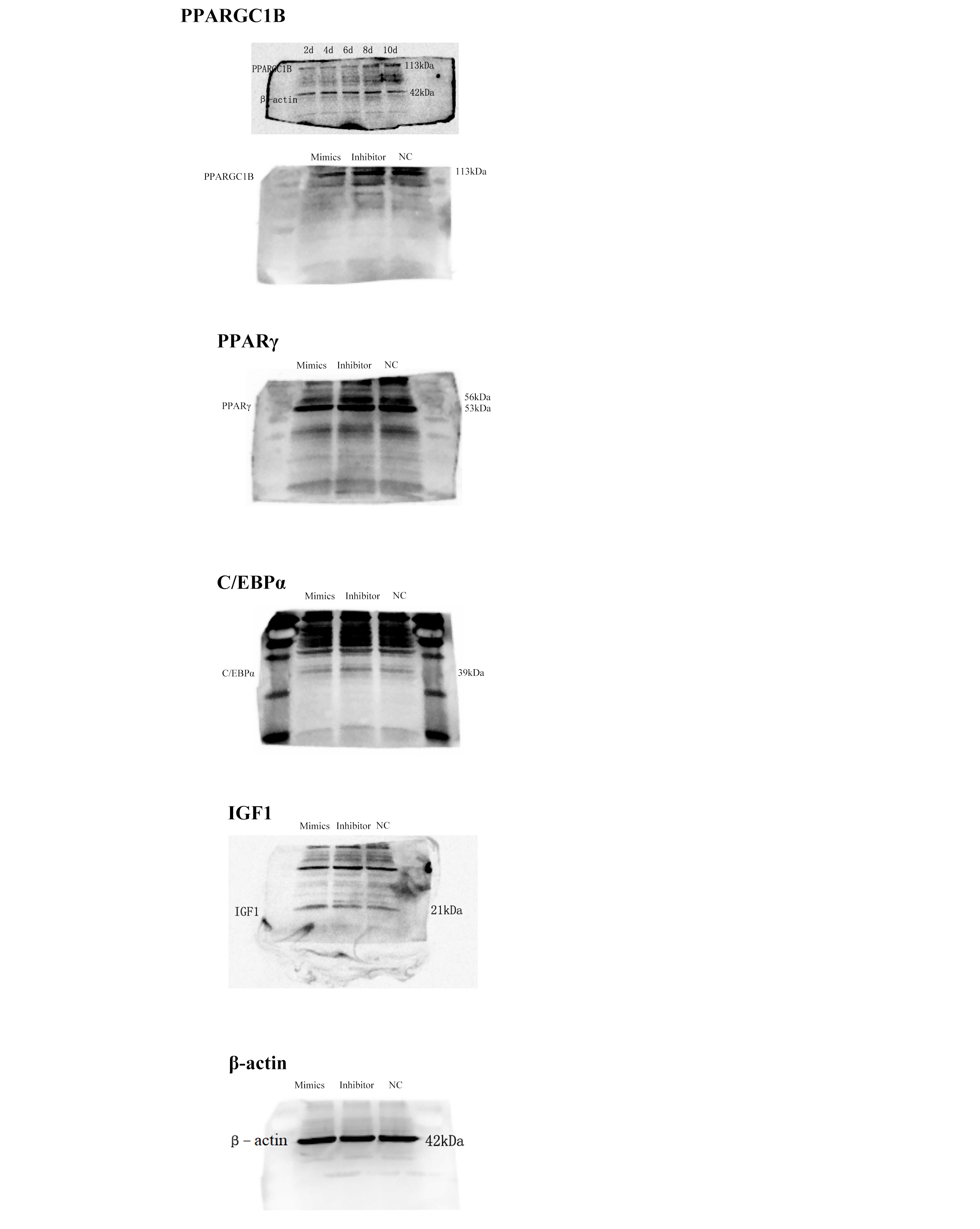

Supplement: Supplemental Material [file KADI_A_2173966_SM4666.tif]
